# ULK1 inhibitor induces spindle microtubule disorganization and inhibits phosphorylation of Ser10 of histone H3

**DOI:** 10.1002/2211-5463.13000

**Published:** 2020-10-26

**Authors:** Xinmiao Ji, Xin Zhang, Zhiyuan Li

**Affiliations:** ^1^ High Magnetic Field Laboratory Key Laboratory of High Magnetic Field and Ion Beam Physical Biology Hefei Institutes of Physical Science Chinese Academy of Sciences Hefei China; ^2^ Institute of Physical Science and Information Technology Anhui University Hefei China

**Keywords:** autophagy, mitosis, MRT68921, polyploidy, SBI‐0206965, ULK1 inhibitor

## Abstract

Certain tumors are dependent on autophagy for survival; thus, the use of unc‐51‐like autophagy activating kinase (ULK) 1 inhibitors to block autophagy has the potential for tumor treatment. However, ULK1 inhibitors affect processes other than autophagy. Herein, we report that the ULK1 inhibitors SBI‐0206965/MRT68921 not only inhibit phosphorylation of histone H3 (Ser10) and delay chromatin condensation but also induce spindle microtubule disorganization to form short and fragmented microtubule polymers. Although the delay in chromatin condensation also delayed mitotic entry, the disorganized microtubule polymers resulted in unsegregated chromosomes and polyploidy. Although the effect on mitotic entry was moderate, polyploidy formation was decreased in ULK1 knockout cells with or without ULK2 knockdown. In conclusion, it will be helpful to consider the roles of ULK1 inhibitors in mitotic dysregulation, as well as autophagy, when evaluating their antitumor efficacy.

## Graphical abstract

Previous reports have demonstrated that SBI‐0206965/MRT68921 inhibit autophagy through unc‐51‐like autophagy activating kinase 1 inhibition. Herein, we report their new function in mitotic dysregulation, mediating spindle microtubule disorganization and phosphorylation of histone H3 (Ser10). This finding may provide insights into tumor treatment with the ULK1 inhibitors.

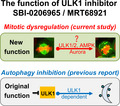

**Abbreviations**
AMPK: AMP‐activated protein kinase
ATG: autophagy‐related
CDK: cyclin‐dependent kinase
DMEM: Dulbecco's modified Eagle's medium
HCT 116: human colorectal cancer
HEK‐293T: human embryonic kidney 293T
IC50: half maximal inhibitory concentration
KO: knockout
mTOR: mammalian target of rapamycin
pH3(S10): phospho‐histone H3 serine 10
PI: propidium iodide
RFP: red fluorescent protein
ULK: unc‐51‐like autophagy activating kinase

Autophagy is a conserved degradation pathway for the elimination of unwanted cellular components, such as damaged proteins, organelles and cytosol. This process is vital in starvation stress for nutrient supply, in cellular and tissue homeostasis during pathogen defense and in various human diseases, such as cancers, neurodegenerative diseases and infectious diseases [1, 2, 3]. Because autophagy could promote cell survival for tumor cells [4, 5], autophagy inhibition has been investigated for tumor inhibition recently [6, 7, 8]. Although chloroquine or hydroxychloroquine as an autophagy inhibitor has entered clinical study [9, 10], it is far from specific due to its effect on lysosome and endosome. Unc‐51‐like autophagy activating kinase (ULK) 1, the mammalian ortholog of Atg1 in yeast, is the only serine–threonine protein kinase in core autophagy machinery. ULK1 and ULK2 share 52% similarity in amino acid and 78% kinase domain, the similar interacting proteins in autophagy regulation [11, 12]. In most cases, such as autophagy and metabolism, ULK1 and ULK2 are reported to function synergistically [12, 13]. ULK1 complex, consisting of ULK1, autophagy‐related (ATG) 13, FAK family‐interacting protein of 200 kDa and ATG101, is upstream of autophagy machinery in which external and internal signals from AMP‐activated protein kinase (AMPK) or mammalian target of rapamycin (mTOR) converge to the downstream autophagy executors, such as VPS34 complex and ATG12–ATG5–ATG16l conjugate [14, 15, 16, 17]. Due to its specific and central role in autophagy, ULK1 has been considered a promising drug target for autophagy inhibition in autophagy‐promoted diseases. ULK1 inhibitors, including SBI‐0206965, MRT68921 and ULK1‐101, were discovered to inhibit ULK1 kinase activity [6, 7, 8]. For example, SBI‐0206965 was reported to be cytotoxic to glioblastoma, neuroblastoma and lung cancer cells [18, 19, 20], and MRT68921 potentiates chemosensitivity in mesothelioma [21] and induces apoptosis and autophagy in FLT3‐ITD‐mutated acute myeloid leukemia [22].

Mitosis undergoes a tremendous change in morphology and structure in only 1–2 h from chromatin condense, nuclear envelope breakdown, spindle formation to chromosome alignment and the nuclear envelope re‐formation [23]. The mitotic progression is tightly controlled, and its dysregulation has been found in many tumors. The blockade of mitotic progression is an ideal approach to induce mitotic catastrophe to kill cancer cells [24, 25]. Taxol, vinblastine and inhibitors of Aurora kinase A/B, cyclin‐dependent kinase 1 (Cdk1), CENP‐E, Eg5, Cdc20 and Polo‐like kinase have been developed to target mitotic progression [24, 25, 26].

Autophagy has been reported to be closely related to cell cycle or mitosis [23, 27, 28, 29], which indicates a potential combinatorial therapy with mitotic drugs for tumors dependent on autophagy for survival. Although autophagy regulators such as ULK1, AMPK, ATG13 and p62 were shown to regulate mitosis [30, 31, 32, 33, 34], whether ULK1/2 inhibitors are involved in mitosis is unknown. Herein we found that ULK1 inhibitors SBI‐0206965 or MRT68921 induced striking spindle microtubule fragmentation and polymerization and chromatin condensation delay, which induced polyploidy formation and mitotic entry delay, respectively. Besides autophagy, our data showed another potential of SBI‐0206965 and MRT68921 in tumor inhibition by dysregulating mitotic progression.

## Materials and methods

### Reagents

SBI‐0206965 was from Selleck (Houston, TX, USA), and MRT68921 was from Sigma (St. Louis, MS, USA). ULK1 antibody (#8054), LC3B antibody (#3868), the cell‐cycle regulation antibody sampler kit II (#9870) and the horseradish peroxidase‐linked anti‐rabbit and anti‐mouse IgG were all from Cell Signaling Technology (Danvers, MA, USA). The anti‐β‐Tubulin, anti‐(glyceraldehyde 3‐phosphate dehydrogenase) and anti‐β‐actin Ig and TransStart FastPfu DNA Polymerase were from Beijing TransGen Biotech (Beijing, China). Nocodazole was from Selleck. Propidium iodide (PI)/RNase staining buffer was from BD Pharmingen (San Diego, CA, USA). The siRNAs were ordered from Genepharm (Shanghai, China), and primers were from Sangon Biotech (Shanghai, China). HiPerFect was from Qiagen (Dusseldorf, Germany). Protease inhibitor and phosphatase inhibitor cocktails were from Roche (Basel, Switzerland), and the polyvinylidene fluoride membrane was from Millipore (Billerica, NJ, USA). GlutaMAX was from Gibco (Carlsbad, CA, USA). The secondary fluorescently conjugated antibodies, Antifade ProLong Gold, were from Molecular Probes (Eugene, OR, USA). M‐MLV Reverse Transcriptase and TRIzol were from Invitrogen (Waltham, MA, USA). Prestained Protein Ladder (26616) and M‐PER were from Thermo Pierce (Waltham, MA, USA). CellTiter‐Glo (#G7570) was from Promega (San Luis Obispo, CA, USA).

### Cell culture and generation of ULK1 knockout cell lines

HeLa, HCT116 and human embryonic kidney 293T (HEK293T) cells were cultured in Dulbecco's modified Eagle's medium (DMEM; without l‐glutamine) (Cellgro, CVR15‐107, Manassas, VA, USA) supplemented with 2 mm GlutaMAX, 10% FBS and 1% penicillin–streptomycin. HeLa cells expressing GFP–Tubulin–red fluorescent protein (RFP)–H2B were a gift of Timothy Mitchison from Harvard Medical School and maintained in DMEM complete medium as described earlier with 500 ng·mL^−1^ G418. ULK1 knockout (KO) HeLa/HEK‐293T cells were constructed as described previously [34]. In brief, after 12 h transfection, HeLa or HEK‐293T cells transfected with the plasmid PX458 subcloned with ULK1–guide RNA were diluted into 0.5 cell/100 μL in a 96‐well plate. The single‐cell clones were subjected to western blot analysis using the ULK1 antibody. The single‐cell clone that could not be detected with the ULK1 antibody was selected as the ULK1 KO cell line.

### Cell‐cycle analysis and phospho‐histone H3 serine 10 detection

Cell‐cycle synchronization was performed as described previously [34]. In brief, cells were plated at 25% confluence 24 h before thymidine block assay. Cells blocked with 2.5 mm thymidine in DMEM for 16 h were released for the indicated time and subsequently progressed through S, G2 and M phases. Alternatively, nocodazole (100 ng·mL^−1^) was combined with thymidine block to arrested cells in prometaphase or pseudo‐prometaphase. Cells were fixed with −20 °C 75% EtOH overnight and stained with PI/RNase staining buffer for 15 min at room temperature. For mitotic index analysis, cells fixed as described earlier were stained with phospho‐histone H3 serine 10 [pH3(S10)] at 1 : 1600 for 2 h at room temperature and washed twice before Alexa 488‐conjugated anti‐rabbit IgG staining. The data were acquired with flow cytometry (CytoFLEX; Beckman Coulter, Brea, CA, USA) and analyzed by modfit lt 4.1 (Topsham, ME, USA) and flowjo VX software (LLC, Ashland, OR, USA).

### RNA interference assay

HeLa or HeLa–ULK1 KO cells were plated in a 12‐well plate, and 40 nm siRNAs for human ULK2 or negative control was transiently transfected using HiPerFect following the manufacturer's protocol. The siRNA sequence for human *ULK2* [35] was *GUGGAGACCUCGCAGAUUA* according to the previous report, and negative control was *UUCUCCGAACGUGUCACGUdTdT* as Gemapharm recommendation. In brief, for each well, 2.4 μL siRNA of 20 μm was mixed with 200 μL Opti‐MEM, and then 6 μL HiPerFect was added into the diluted siRNA and mixed well with vortexing. After incubation for 20 min at room temperature, the siRNA–HiPerFect complex was added dropwise into cells followed by gently swirling the plate to ensure uniform distribution of the transfection complex. After 24‐h siRNA transfection with HiPerFect as the manufacturer's instruction, the cells were synchronized by thymidine and released with or without ULK1 inhibitor treatment. After the indicated time, the cells were either lysed for western blot or fixed to perform flow cytometry or immunofluorescence analysis.

### Semiquantitative RT‐PCR

RNAs were isolated from HeLa or HeLa ULK1 KO cells transfected with siRNA for negative control, or *ULK2* using TRIzol and 500 ng RNA was used in cDNA synthesis reaction by M‐MLV Reverse Transcriptase with random primers and oligo dT. Semi‐quantitative RT‐PCR was performed by TransStart FastPfu DNA Polymerase in a 20‐μL reaction system. The primers used for semiquantitative RT‐PCR were as follows: ULK2‐F, GCCAGAAAACTGATTGGGAGGTAGC and ULK2‐R, ATCGTGTCTTCACTGAGAGTCCCTT; β‐Actin‐F, CCAACCGCGAGAAGATGA and β‐Actin‐R, CCAGAGGCGTACAGGGATAG. The programs were run as follows: 95 °C, 3 min; 30 cycles for (95 °C, 20 s; 60 °C, 30 s; 72 °C, 20 s); and 72 °C, 10 min.

### Live‐cell imaging of HeLa cells expressing GFP–Tubulin–RFP–H2B

HeLa–GFP–Tubulin–RFP–H2B cells seeded into the glass‐bottom six‐well cell culture (Nest Biotech, Wuxi, China) were synchronized by thymidine release and treated with DMSO, 10 μm SBI‐0206965 or 10 μm MRT68921. The live cells at 37 °C, 5% CO_2_ incubator were then subjected to live‐cell imaging by the Nikon Ti2‐E‐W1 spinning disk equipped with Plan 20× Apochromat Lambda objective (Nikon Corp., Tokyo, Japan) using the 488 and 561 channels at 10‐min intervals for 50 h. The images were processed by Nikon NIS‐ELEMENTS AR software.

### Immunofluorescence and western blot

The detailed procedure was described previously [36]. In brief, cells grown on coverslips were fixed with −20 °C methanol for 5 min and subsequently subjected to immunofluorescence using β‐Tubulin antibody and Alexa 488‐conjugated anti‐mouse IgG. Cells were then stained with 300 nm DAPI (4′,6‐diamidino‐2‐phenylindole) at room temperature for 2 min and mounted with Antifade ProLong Gold. The immunofluorescence images were captured by Nikon Ti2‐E‐W1 spinning disk equipped with Plan 20× Apochromat Lambda objective or Leica DMI4000 B fluorescent microscope (Leica Camera, Wetzlar, Germany). The whole‐cell lysate from cells lysed by M‐PER was mixed with SDS loading buffer and denatured at 95 °C for 7 min. The samples were subjected to the SDS/PAGE and western blot, and the results were obtained by Tanon Fine Do X6 (Shanghai, China).

## Results

### SBI‐0206965/MRT68921‐induced spindle microtubule disorganization and pH3(S10) inhibition resulted in prolonged mitotic duration and chromatin condensation delay

**Fig. 1:**
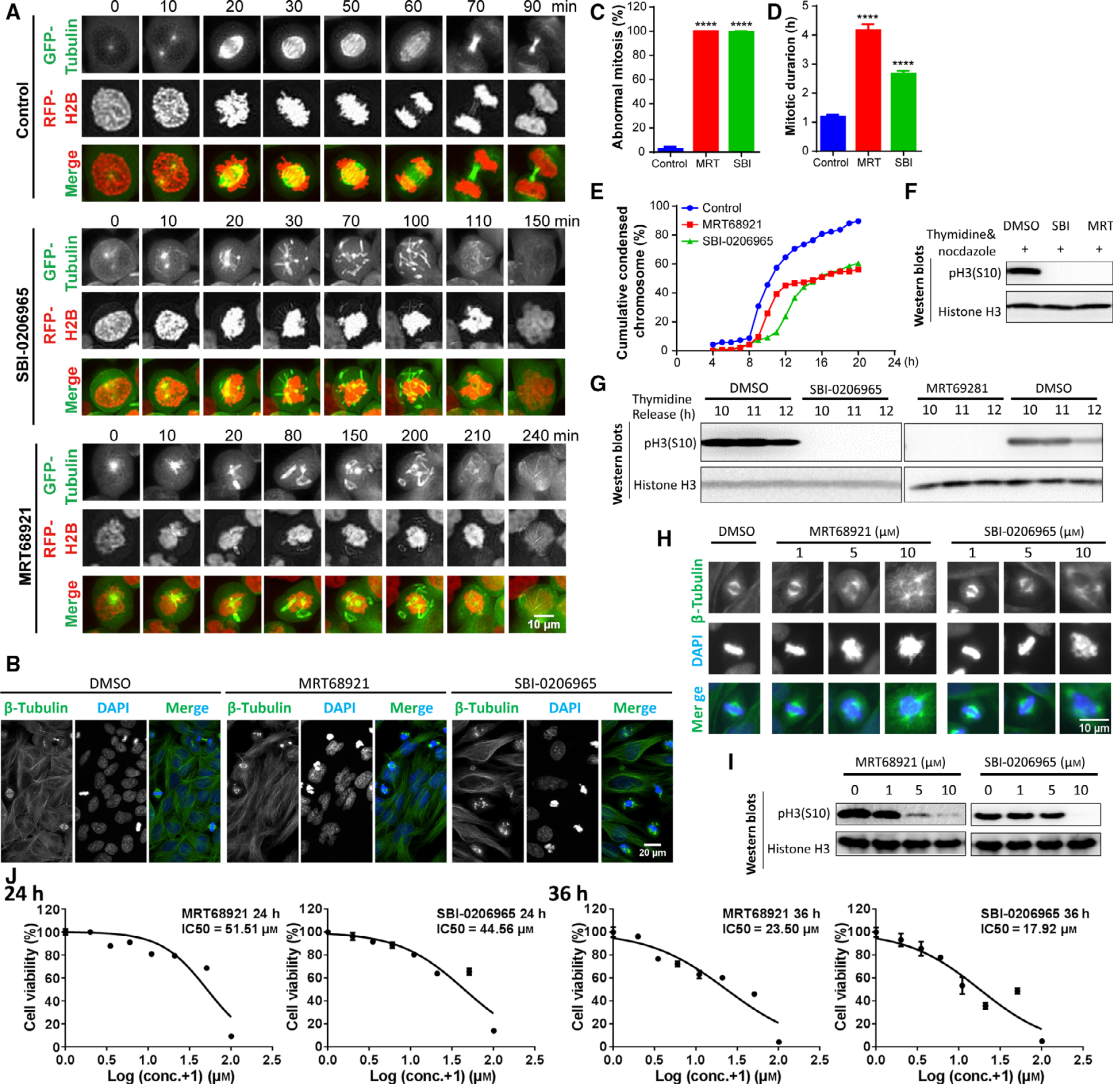
SBI‐0206965/MRT68921‐induced spindle microtubule disorganization and pH3(S10) inhibition result in prolonged mitotic duration and chromatin condensation delay. (A) Microtubules are significantly tubulated to form short and thick tubules that quickly shuttled randomly when treated with 10 μm SBI‐0206965 or MRT68921. HeLa cells stably expressing GFP–Tubulin and RFP–H2B released from thymidine were treated with SBI‐0206965/MRT68921 for live‐cell imaging detection using Z‐stack every 10 min. The maximum intensity projection micrographs were analyzed by the automatic deconvolution method and imagej (NIH, Bethesda, MD, USA). Scale bar, 10 μm. (B) SBI‐0206965/MRT68921‐induced microtubule disorganization. HeLa cells released from thymidine were treated with SBI‐0206965/MRT68921 for 13 h and analyzed by immunostaining by β‐Tubulin and DAPI. Scale bar, 20 μm. (C) The percentage of abnormal mitosis. Cells were treated as in (A), and the data were from three independent statistical analyses. ****P < 0.0001. (D) The mitotic duration. Cells were treated as in (A), and the data were from statistical analysis with *n* = 50 in each group. ****P < 0.0001. Student’s *t*‐test was used for statistical analysis, and data are shown as mean ± standard error of the mean (C, D). (E) The chromatin condensation delay. According to the live‐cell imaging, we recorded the condensed chromosomes with a morphological change from the long, thin chromatin in interphase to prometaphase/metaphase chromosome. The data were from (A). (F, G) SBI‐0206965 or MRT68921 abolished pH3(S10) in thymidine‐released cells with or without nocodazole arrest. HeLa cells in mitosis synchronized by thymidine release and nocodazole arrest were treated with SBI‐0206965/MRT68921 for 13 h (F), and HeLa cells released from thymidine were treated with SBI‐0206965 and MRT68921 for the indicated time (G). Cells were then subjected to western blot analysis. (H, I) Titration of SBI‐0206965/MRT68921 for pH3(S10) inhibition and abnormal mitosis. HeLa cells released from thymidine were treated with 0, 1, 5 and 10 μm SBI‐0206965/MRT68921 for 12 h and analyzed using immunostaining by β‐Tubulin, DAPI and western blot. Scale bar, 10 μm. (J) The IC50 of the cytotoxic threshold of SBI‐0206965 and MRT68921. HeLa cells were treated with 0–100 μm SBI‐0206965/MRT68921 for 24 and 36 h, respectively, and cell viability was determined by CellTiter‐Glo. The data were acquired with the Multimode Plate Reader (EnVision; PerkinElmer) and analyzed by graphpad prism 6 (GraphPad Software, La Jolla, CA, USA).

Autophagy regulators were shown to modulate mitosis [30, 31, 32, 33, 34], but the effect of ULK1 inhibitor on mitosis is unknown, which hampered the comprehensive understanding of ULK1 inhibitor’s therapeutic potential. HeLa cells stably expressing GFP–Tubulin and RFP–H2B released from thymidine were treated with the widely used ULK1 inhibitors, SBI‐0206965 or MRT68921, to examine their effects on mitosis. According to live‐cell imaging, although nuclear envelope breakdown could occur, the centrosome could not move to the poles of the cell, which potentially disrupted subsequent mitotic spindle dynamics and chromosome alignment. During this stage, SBI‐0206965 or MRT68921 induced mitotic microtubules to form short and fragmented polymers that quickly shuttled around the chromosomes (Fig. 1A and Videos [Link], [Link], [Link]). These disorganized spindle microtubules were present in nearly 100% of cells under mitosis, even if microtubules in nonmitotic cells did not change significantly (Videos [Link], [Link], [Link]). This similar phenotype was also detected by immunostaining of wild‐type HeLa cells with anti‐Tubulin antibodies (Fig. 1B). In addition, SBI‐0206965 or MRT68921 induced significantly increased abnormal mitosis and prolonged mitotic duration (Fig. 1C,D). Because chromosome condensation occurs in mitosis accompanying the dramatic morphological change from the long, thin chromatin in interphase to prometaphase–metaphase chromosome [37], we found that cells treated with the ULK1 inhibitor could enter prophase, but the simultaneous chromatin condensation was delayed (Fig. 1E). PH3(S10), one of the aurora B’s substrates [38], was tightly correlated with chromosome condensation that is important for mitotic progression. Both inhibitors almost abolished pH3(S10) level (Fig. 1F,G). To confirm the lowest effective concentration, we titrated SBI‐0206965/MRT68921 for pH3(S10) inhibition and abnormal mitosis in HeLa cells. The 10 μm SBI‐0206965 or MRT68921 would be effective for either complete pH3(S10) inhibition or abnormal mitosis and were used in this article unless otherwise indicated (Fig. 1H,I). We also tested the half maximal inhibitory concentration (IC50) of the cytotoxic threshold of SBI‐0206965 and MRT68921 for HeLa cells to interpret whether mitotic disorganization is possibly due to apoptosis. The IC50 of MRT68921 is 51.51 and 23.5 μm and that of SBI‐0206965 is 44.56 and 17.92 μm when treated for 24 and 36 h, which is higher than the 10 μm used in this article (Fig. 1J), suggesting that mitotic disorganization is unlikely due to apoptosis. These striking phenotypes of ULK1 inhibitor on spindle microtubule disorganization and pH3(S10) inhibition have not been reported before, which prompted us to further detect their potential consequences.

### SBI‐0206965‐ and MRT68921‐induced polyploidy

**Fig. 2:**
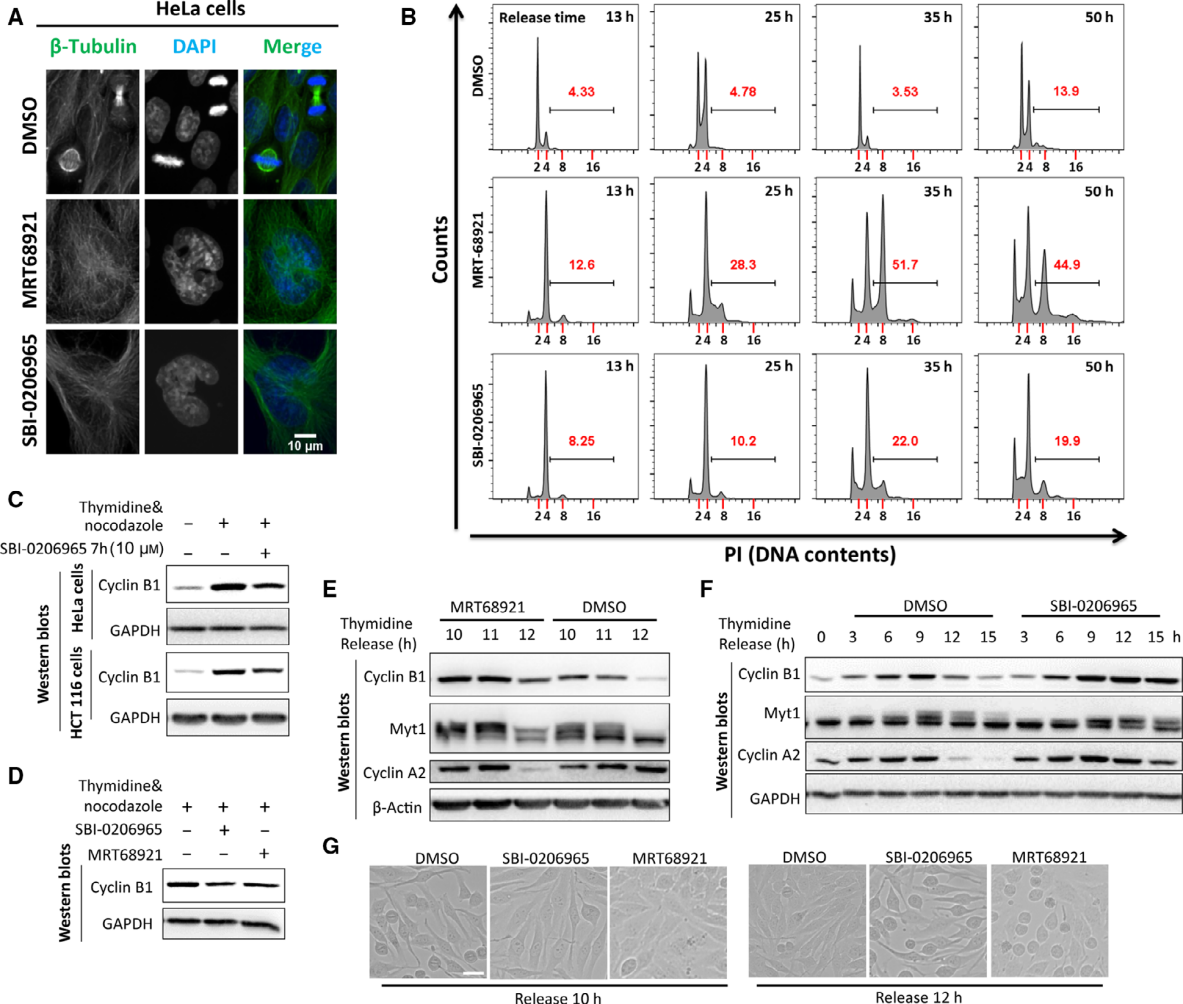
SBI‐0206965‐ and MRT68921‐induced polyploidy and mitotic entry delay. (A) Multinucleated cells or polyploidy formed when treated by SBI‐0206965 or MRT68921. HeLa cells released from thymidine block were treated with 10 μm SBI‐0206965 or MRT68921 for 35 h and analyzed by immunostaining by β‐Tubulin and DAPI. Scale bar, 10 μm. (B) SBI‐0206965‐ or MRT68921‐induced polyploidy. HeLa cells treated as in (A) were fixed with −20 °C 75% EtOH overnight and analyzed by PI staining and flow cytometry. The numbers in red are the percentage of polyploidy induced by SBI‐0206965 or MRT68921. The bar defines the percentage of cells with polyploidy, indicating cells with more than 4N of DNA. (C) SBI‐0206965 inhibited cyclin B1 accumulation during mitosis. HeLa or HCT 116 cells synchronized into mitosis by thymidine release and subsequent nocodazole arrest were treated with ULK1 inhibitor SBI‐0206965 for 7 h. (D) MRT68921 inhibited cyclin B1 accumulation as SBI‐0206965. HeLa cells synchronized as in (C) were treated with SBI‐0206965 or MRT68921. (E, F) SBI‐0206965/MRT68921 delayed mitotic entry and cell‐cycle progression. HeLa cells released from thymidine block were treated with SBI‐0206965/MRT68921 for the indicated time and were analyzed by various cell‐cycle markers. (G) SBI‐0206965/MRT68921 delayed mitotic entry. HeLa cells treated as in (E, F) were delayed in mitotic entry. Scale bar, 20 μm.

Due to the striking disorganization in the spindle microtubule, we first seek to examine the consequences. Using thymidine release and immunofluorescence, we found that multinucleated cells or polyploidy formed when HeLa cells were treated by SBI‐0206965 or MRT68921 (Fig. 2A). To accurately quantify the percentage of polyploidy, we used flow cytometry to assess the DNA contents. When treated by SBI‐0206965 or MRT68921 for 13 h from thymidine release, most cells were blocked in the G2/M phase shown by the 4N DNA content (a 4N peak in the flow cytometry, cells in G1 phase with a 2N peak in the flow cytometry). During 25–35 h of treatment, the percentage of polyploidy containing 8N DNA increased (Fig. 2B). The cell size was also increased when cells were treated by the ULK1 inhibitor (Fig. 2A), which was consistent with the increased DNA content from 2N to 4N/8N.

### SBI‐0206965 and MRT68921 delayed mitotic entry

Because the pH3(S10) is vital for chromatin condensation and mitotic entry [38], we next analyzed the effect of the ULK1 inhibitor on mitotic entry. Cells synchronized by thymidine release and nocodazole arrest were treated with ULK1 inhibitor SBI‐0206965. In both HeLa and human colorectal cancer (HCT 116) cells, SBI‐0206965 decreased the level of cyclin B1 that forms an active complex with CDK1 to initiate mitotic entry (Fig. 2C,D). Besides, MRT68921, another ULK1 inhibitor, also decreases the cyclin B1 level as SBI‐0206965 (Fig. 2D). Considering that cyclin B1 is the functional and structural binding partner for CDK1 and its protein level accumulated from late G2 to metaphase [39], the decreased cyclin B1 level indicates that the mitotic entry is delayed. In mitosis, cyclin A is also up‐regulated and myelin transcription factor 1 has an upshift band [40]. To rule out the side effect of nocodazole, thymidine‐released HeLa cells were treated with SBI‐0206965 or MRT68921, respectively. Similarly, mitotic entry and cell‐cycle progression are delayed when treated by SBI‐0206965 or MRT68921 according to cell‐cycle markers such as cyclin B1 and myelin transcription factor 1, cyclin A and p‐Cdc2(Y15) (Fig. 2E,F). In the thymidine release group, cells enter mitosis and early G1 phase (Fig. 2E, right panel; Fig. 2F, left panel; Fig. 2G, ‘DMSO’ subgroup). In the thymidine release group for 10 h, most DMSO‐treated cells begin to enter mitosis and become round from flat state in interphase, whereas cells treated with ULK1 inhibitors remain flat. ULK1 inhibitor‐treated cells become round until thymidine release for 12 h (Fig. 2G), which indicates the delayed mitotic entry when cells were treated with either ULK1 inhibitor.

### ULK1 and ULK2 differentially regulate SBI‐0206965/MRT68921‐induced polyploidy and mitotic entry delay

Given that SBI‐0206965 and MRT68921 were mainly recognized as ULK1 inhibitors and the high similarity between ULK1 and ULK2, we next aimed to investigate the role of ULK1 and ULK2 in polyploidy and mitotic entry delay.

**Fig. 3:**
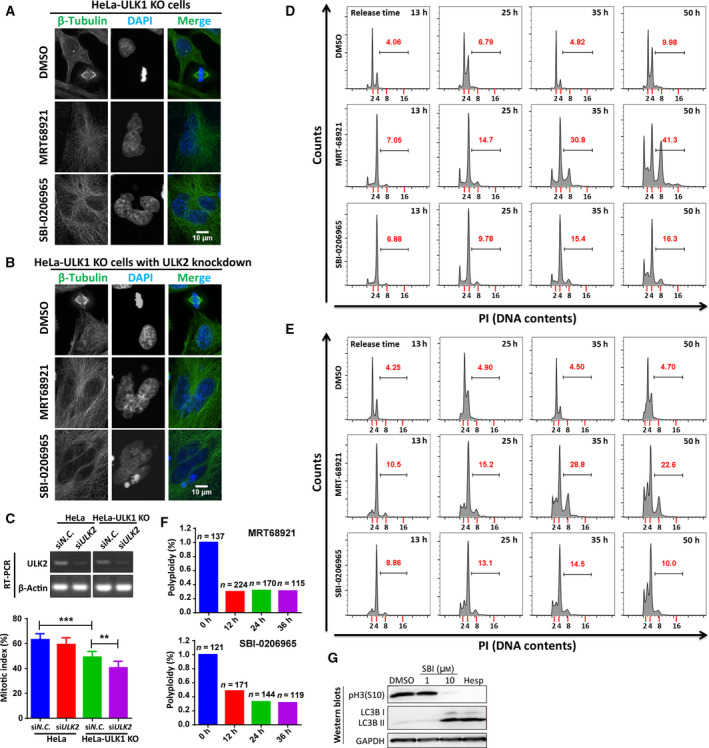
ULK1/2 contributes to SBI‐0206965‐ or MRT68921‐induced polyploidy. (A, B) Polyploidy induced by SBI‐0206965 or MRT68921 in HeLa–ULK1 KO cells or HeLa–ULK1 KO cells with ULK2 knockdown. Cells released from thymidine block were treated with 10 μm SBI‐0206965 or MRT68921 for 35 h and analyzed by immunostaining by β‐Tubulin and DAPI. Scale bars, 10 μm. (C) ULK1 KO and/or ULK2 knockdown decreased the mitotic index. HeLa/HeLa ULK1 KO cells were transfected with siRNAs for negative control (N.C.) or *ULK2* and analyzed by RT‐PCR for knockdown efficiency or by flow cytometry for detection of pH3(S10) in thymidine release and nocodazole‐arrested mitosis. Student's *t*‐test was used for statistical analysis, and data are shown as mean ± standard error of the mean. *n* = 3; **P < 0.01, ***P < 0.001. (D, E) SBI‐0206965‐ or MRT68921‐induced differential polyploidy. The numbers in red are the percentage of polyploidy induced by SBI‐0206965 or MRT68921. The bars define the percentage of cells with polyploidy, indicating cells with more than 4N of DNA. Cells treated as in (A, B) were fixed with −20 °C 75% EtOH overnight for PI staining and analyzed by flow cytometry and flow jo. (F) The polyploidy when cells are transitioned to fresh medium during 0–36 h. HeLa cells synchronized with thymidine release and nocodazole arrest were treated with 10 μm SBI‐0206965 or MRT68921 before release into drug‐free fresh medium. Then cells were analyzed by immunostaining of β‐Tubulin and DAPI, and multinucleated cells (polyploidy) were counted and quantified. (G) Comparison of the effect of SBI‐0206965 (SBI) and Hesperadin (Hesp) on autophagy and pH3(S10) inhibition. HeLa cells were treated with 100 nm Hesperadin or 10 μm SBI‐0206965 for 30 h and analyzed by western blot.

We found that SBI‐0206965 and MRT68921 induced polyploidy in HeLa ULK1 KO cells with or without ULK2 knockdown by siRNA as HeLa cells (Fig. 3A,B). Because there is no commercially available ULK2 antibody, we characterized the siRNA of ULK2 knockdown efficiency by RT‐PCR in both wild‐type and ULK1 KO HeLa cells (Fig. 3C, upper panel) and also found that ULK1 KO and/or ULK2 knockdown cells decreased the mitotic index compared with wild‐type cells (Fig. 3C, lower panel). Compared with HeLa cells, ULK1 KO with or without ULK2 knockdown delayed and decreased the polyploidy formation (Figs 2B and 3D,E). For example, MRT68921 treatment for 35 h induced 51.7% of HeLa cells with 8N DNA content (Fig. 2B, middle panels), compared with that of 30.8% of ULK1 KO cells and 28.8% of ULK1 KO cells with ULK2 knockdown (Fig. 3D,E, middle panels). Similarly, when treated by SBI‐0206965, the percentage of HeLa cells with 8N DNA content is also more than that of ULK1 KO cells or ULK1 KO cells with ULK2 knockdown cells (Fig. 2B, lower panels; Fig. 3D,E, lower panels). The data showed that ULK1 and ULK2 are involved in the polyploidy formation. Further, we induced polyploidy in HeLa cells using SBI‐0206965 or MRT68921 and then transitioned the cells to fresh growth media in a time‐course experiment. According to the quantification from immunofluorescence, the polyploidy was significantly decreased when cells were released into fresh media during the time frame of 0–36 h (Fig. 3F). To connect the role of ULK1/2 in polyploidy to their original roles in autophagy, we compared SBI‐0206965 and Hesperadin, a highly selective Aurora kinase inhibitor [41], and found that aurora kinase inhibition by Hesperadin induced autophagy marker LC3B increase (Fig. 3G), indicating the possible effect of autophagy on the mitotic phenotype. Also, SBI‐0206965 induced pH3(S10) inhibition as Hesperadin did (Fig. 3G). The data here indicate a possible effect of autophagy on the mitotic phenotype, which is consistent with our previous finding [34].

**Fig. 4:**
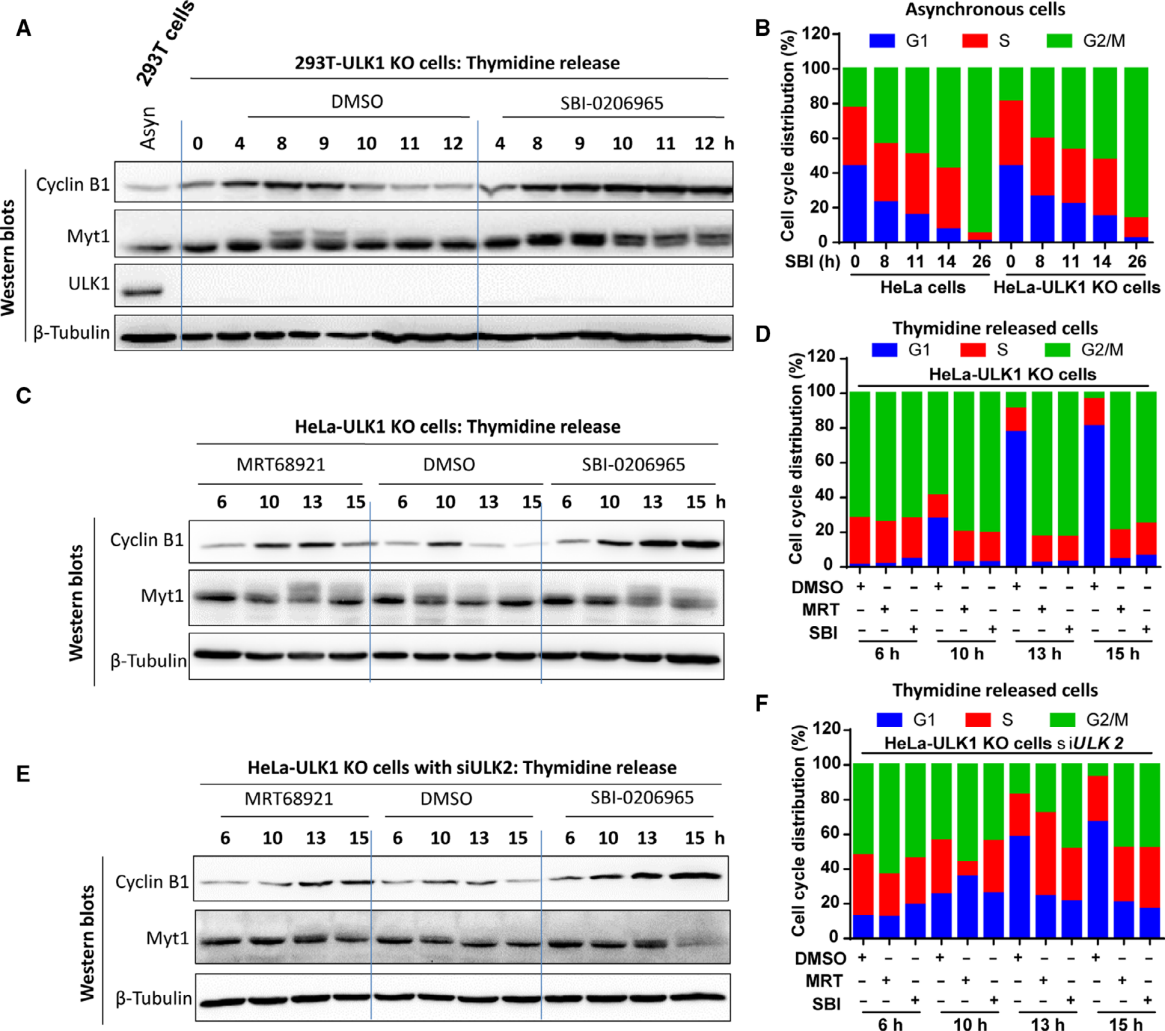
SBI‐0206965‐ or MRT68921‐induced mitotic entry delay is moderately affected by ULK1/2. (A) SBI‐0206965‐induced mitotic entry delay in ULK1 KO 293T cells. 293T ULK1 KO cells released from thymidine were treated with SBI‐0206965 for the indicated time and lysed for western blot analysis. (B) SBI‐0206965‐induced G2/M accumulation in asynchronous HeLa to HeLa‐ULK1 KO cells. Asynchronous HeLa or HeLa‐ULK1 KO cells were treated with SBI‐0206965 for the indicated time and fixed with −20 °C 75% EtOH for PI staining and cell‐cycle distribution analysis by flow cytometry. (C, D) SBI‐0206965/MRT68921 could induce mitotic entry delay and delayed G2/M accumulation in HeLa‐ULK1 KO cells. HeLa‐ULK1 KO cells released from thymidine block were treated with SBI‐0206965/MRT68921 for the indicated time and subjected to western blot and cell‐cycle distribution analysis. (E, F) SBI‐0206965/MRT68921 could induce mitotic entry delay and delayed G2/M accumulation in HeLa‐ULK1 KO cells with ULK2 knockdown. HeLa‐ULK1 KO cells with ULK2 knockdown were treated and analyzed as in (C, D).

Furthermore, to confirm the roles of ULK1 in SBI‐0206965‐ or MRT68921‐induced mitotic delay, we also used ULK1 KO cells. Although SBI‐0206965 treatment could induce mitotic entry delay shown by mitotic markers in thymidine‐released HEK‐293T KO cells (Fig. 4A), we further compared the cell‐cycle distribution between wild‐type and ULK1 KO cells. In asynchronous HeLa and HeLa–ULK1 KO cells, SBI‐02026965 induced similar cell‐cycle distribution and G2/M accumulation (Fig. 4B). Because ULK2 has a similar structure and function to ULK1 [12], the role of ULK2 in mitotic entry delay was further examined. In thymidine‐released cells, although SBI‐0206965 or MRT68921 treatment could still induce mitotic entry delay in ULK1 KO cells with or without ULK2 knockdown, the extent of the delay was moderately affected by both ULK1 KO and ULK2 knockdown compared with ULK1 KO alone as shown by mitotic markers (Fig. 4C,E). Moreover, the G2/M accumulation was delayed by both ULK1 KO and ULK2 knockdown compared with ULK1 KO alone (Fig. 4D,F), indicating that both ULK1/2 contribute to ULK1 inhibitor‐induced G2/M accumulation. For example, when released from thymidine for 10 h, SBI‐0206965‐ or MRT68921‐induced G2/M accumulation was even antagonized by ULK1 KO and ULK2 knockdown compared with ULK1 KO alone (Fig. 4D,F, the ‘10 h’ subgroup).

## Discussion

Autophagy has been known to function in numerous diseases, such as tumors and neurodegeneration diseases. Up to now, ULK1 as a potential target for autophagy activity regulation has been reported in a large amount of the literature, and ULK1 inhibitors have been reported to function in lung cancer cells, FLT3‐ITD‐mutated acute myeloid leukemia and neuroblastoma [8, 19, 20, 22]. However, two ULK1 inhibitors, SBI‐0206965 and MRT68921, that we investigated here induced striking microtubules polymerization and abolished pH3(S10) level to dysregulate mitosis, in which ULK1/2 was differentially involved. Because we recently found that the ULK1–ATG13 complex orchestrated the cell‐cycle progression and the ULK1 kinase activity is not required for mitotic entry [34], the mitotic entry delay induced by the ULK1 inhibitor that inhibits ULK1 kinase activity may result from the side effect rather than ULK1/2 kinase activity.

During normal cell division, bipolar attachment of all chromosomes to mitotic spindle ensures sister chromatid segregation. When cells were treated with SBI‐0206965/MRT68921, we observed abnormal chromosome segregation induced by multipolar spindles, resulting in cytokinesis failure and binucleated cell formation. If the binucleated cells could escape cell death to continue their cell‐cycle progression into another round of mitosis, the multinucleated cells form. Alternatively, the binucleated or multinucleated cells might undergo cell death during either ULK1 inhibitor treatment (Videos [Link], [Link], [Link]). As to SBI‐0206965, most binucleated cells underwent cell death before entering the second round of mitosis according to the live‐cell imaging. However, the binucleated cells induced by MRT68921 could continue their cell‐cycle progression into the second round of mitosis (Videos [Link], [Link], [Link]), which was consistent with the higher percentage of polyploidy in MRT68921‐treated cells compared with SBI‐0206965‐treated cells. Besides, when these induced polyploid cells are transitioned to fresh growth media, the polyploidy percentage is decreased. The ultimate fate of the stably maintained polyploidy needs to be further investigated in future work.

According to previous reports of kinase profiling screen for SBI‐0206965 and MRT68921 [6, 7, 42], their potential target included mitotic kinases aurora A and/or aurora B, which are essential for centrosome maturation and separation, chromosome segregation and cytokinesis, respectively [29, 43]. The inhibition or depletion of aurora B abolished pH3(S10), which could delay mitotic entry [41]. ULK1/2 inhibitor‐induced pH3(S10) inhibition is similar to that of aurora B kinase inhibitor, Hesperadin [41]. In addition, the effect of the current ULK1/2 inhibitors on mitotic entry delay is similar to that of aurora B inhibition [41]. Given that MRT68921 or SBI‐0206965 inhibited aurora A [7, 42], the centrosome could not be well separated in mitotic cells treated by the ULK1 inhibitor.

Besides, the potential target for both inhibitors related to mitosis may be AMPK that is the target for SBI‐0206965 or MRT68921 [7, 44] and functions in mitosis [30, 31]. Due to more targets of both inhibitors [6, 7, 42, 44], their substantial effects on mitosis may be because of the combinatorial effect of more targets, such as aurora kinases and AMPK. Given these two chemicals induced polyploidy, but with fewer rates of polyploidy without ULK1/2, the other kinases may be the major players in this phenotype. Interestingly, in Fig. 2B, when the control cells were treated with DMSO for 50 h, the likelihood of polyploidy is larger than less incubation time and ULK1/2‐deficient cells treated with DMSO for 50 h. Two reports [45, 46] showed that nutrient starvation also blocks the onset of mitosis in mammalian cells and the fission yeast *Schizosaccharomyces pombe*, which indicated that nutrient starvation will induce cells in the G2/M border. Likewise, after 50‐h incubation of cells in the growth medium, the nutrient was deficient and cells were in starvation. Hence relatively more cells are trapped in the G2/M phase, which accumulated more polyploidy than less time incubation. Because ULK1 KO HeLa cells with or without ULK2 knockdown decreased the mitotic index compared with wild‐type cells, such cells grow slower with prolonged cell cycle than wild‐type cells, which ultimately decreased the accumulation of polyploidy as shown in the DMSO control of Figs 2B and 3D,E.

In our opinion, the inhibition of SBI‐0206965 or MRT68921 on the tumor is likely to be orchestrated by a series of targets, including ULKs, aurora kinases and AMPK. Besides the autophagy role, when evaluating the ULK1/2 inhibitors’ antitumor efficacy, it will be helpful to take into account the contribution of their roles in mitotic dysregulation. In the future, the development of ULK kinase inhibitors with improved selectivity for autophagy inhibition remains for further investigation.

## Conflict of interest

The authors declare no conflict of interest.

## Author contributions

ZL and XJ performed the experiments. ZL and XZ designed the experiments, analyzed data and wrote the paper. All authors edited the manuscript.

## Supporting information


**Video S1.** Live‐cell imaging for HeLa cells stably expressing GFP–Tubulin and RFP–H2B treated by DMSO.

**Video S2.** Live‐cell imaging for HeLa cells stably expressing GFP–Tubulin and RFP‐H2B treated by SBI‐0206965.

**Video S3.** Live‐cell imaging for HeLa cells stably expressing GFP–Tubulin and RFP–H2B treated by MRT68921. Cells released from thymidine for 3 h were treated with DMSO, SBI‐0206965 and MRT68921, respectively. The movies were captured by Nikon Ti2‐E‐W1 spinning disk equipped with Plan 20X Apochromat Lambda objective using the 488 and 561 channels at 10‐min intervals. The movie length is 48 h and 40 min.

## Data Availability

The data will be available from the corresponding author upon reasonable request.
